# Analysis of Missed Skeletal Injuries Detected Using Whole-Body Bone Scan Applied to Trauma Patients: A Case–Control Study

**DOI:** 10.3390/diagnostics13111879

**Published:** 2023-05-27

**Authors:** Jae Sik Chung, Sanghyun An, Seong Chan Gong, Pil Young Jung

**Affiliations:** 1 Department of Traumatology, Department of Surgery, Wonju Severance Christian Hospital, Yonsei University Wonju College of Medicine, Wonju 26426, Republic of Korea; gsjaesik@yonsei.ac.kr; 2 Department of Surgery, Wonju Severance Christian Hospital, Yonsei University Wonju College of Medicine, Wonju 26426, Republic of Korea; uldura@yonsei.ac.kr (S.A.); surgeon_g@yonsei.ac.kr (S.C.G.)

**Keywords:** whole-body bone scan, missed injuries, multiple traumas, trauma center

## Abstract

(1) Background: Skeletal injuries may be missed in patients presenting multiple traumas during initial assessment. A whole-body bone scan (WBBS) may aid the detection of missed skeletal injuries, but the current level of research in this regard is insufficient. Thus, this study aimed to investigate whether a WBBS is useful for the detection of missed skeletal injuries in patients with multiple traumas. (2) Methods: This retrospective, single-region, trauma center study was conducted at a tertiary referral center from January 2015 to May 2019. The rate of missed skeletal injuries detected via WBBSs was evaluated, and factors that could influence the outcome were analyzed and divided into missed and not-missed groups. (3) Results: A total of 1658 patients with multiple traumas who underwent WBBSs were reviewed. In the missed group, the percentage of cases with an Injury Severity Score (ISS) ≥ 16 was higher than the not-missed group (74.66% vs. 45.50%). The rate of admission route through surgery and embolization was high in the missed group. Moreover, the proportion of patients that experienced shock in the missed group was higher than that in the not-missed group (19.86% vs. 3.51%). In univariate analysis, ISS ≥ 16, admission route through surgery and embolization, orthopedic surgery involvement, and shock were related to missed skeletal injuries. ISS ≥ 16 was determined to be statistically significant in multivariate analysis. Additionally, a nomogram was constructed based on multivariable analysis. (4) Conclusions: Missed skeletal injuries were significantly associated with several statistical factors, and a WBBS can be used as a screening method to detect missed skeletal injuries in patients with multiple blunt traumas.

## 1. Introduction

Skeletal injuries may be missed in patients presenting with multiple traumas during an initial assessment. To avoid missing injuries, the Advanced Trauma in Life Support course proposes the implementation of a head-to-toe secondary survey after the primary survey for life-threatening injuries [1]. Moreover, if needed, a tertiary survey can also help reduce the number of missed diagnoses [2]. However, missed skeletal injuries in multiple-trauma patients are often found later on despite appropriate imaging, history documentation, and physical examinations. The failure to note fractures is influenced by various factors such as inadequate clinical information, the poor general condition of a patient, severe pain, the proficiency of the treating physician, and image quality [3,4,5]. These missed skeletal injuries can have negative impacts, such as increased morbidity, prolonged hospital stays, and increased medical costs, and might precipitate medico-legal issues [6].

A whole-body bone scan (WBBS), a functional imaging modality used to detect metastatic bone tumors, metabolic bone disease, traumatic injuries, and inflammation, might aid the detection of skeletal injuries that may be missed in multiple-trauma patients during an initial evaluation [7]. However, there is little evidence supporting the utility and efficacy of such a scan.

In this study, by reviewing data of patients who had undergone WBBSs at a regional trauma center in a tertiary referral center, we aimed to investigate the usefulness of WBBSs for the detection of missed skeletal injuries and evaluate the factors related to these injuries. The hypothesis of the study was that a WBBS might help detect missed skeletal injuries in multiple-trauma patients.

## 2. Materials and Methods

This study was a retrospective, single-region, trauma center study conducted at a tertiary referral center in South Korea from January 2015 to May 2019. This study was approved by the Institutional Review Board (CR319105) and registered in a Clinical Research Information System that is affiliated with the World Health Organization’s International Clinical Trials Registry Platform (KCT0004371).

### 2.1. Population

During the study period, we reviewed data of patients with multiple traumas who underwent WBBS and divided them into two groups (missed injury group and non-missed injury group) based on the presence or absence of missed diagnoses. The initial diagnoses were made using plain radiography, ultrasonography, and computed tomography (CT) to examine the regions of interest based on patients’ symptoms and physical examinations. If a patient complained of pain or had confirmed injuries in two or more areas of the body, including their internal organs, they were classified as a multiple-trauma patient. The following factors were collected and reviewed: patient’s basic clinical information; Injury Severity Score (ISS); Glasgow Coma Scale (GCS) score; the admission department in which the patient was received, and the duration from admission to evaluation of WBBS; admission route; intensive care unit (ICU); ward; surgery; and embolization. Moreover, deployment of the trauma team (yes or no), involvement of the department of orthopedic surgery (OS) (yes or no), shock state, mechanism of injury, and WBBS results were also reviewed. In addition, we compared and analyzed the incidence of missed skeletal injuries by trauma site and vector and identified additional treatments for missed injuries. Damaged areas were categorized into the face, chest, upper extremity, lower extremity, and spine. Trauma vectors were classified as passenger traffic accident (TA), motorcycle TA, pedestrian TA, workplace accident, fall, and others. Additional types of treatment were classified as surgery, pain control, and immobilization.

### 2.2. Trauma Team Deployment

A trauma team is a group of medical staff that remains at a regional trauma center for 24 h, mimicking the US level 1 trauma center. In this study, the trauma team was composed of experts in general surgery, thoracic surgery, and emergency medicine. Depending on the condition of the severe trauma patients, the mechanism of injury, and emergency ultrasonography results, the trauma team was deployed, and the trauma team’s staff began to treat the patient promptly.

### 2.3. Admission Route

After the trauma team has been deployed and the initial assessment of trauma patients has been completed, the process proceeds to the next step, acting as a definite management plan. The patients were hospitalized for nonoperative or surgical treatment or angiographic embolization. Cases of nonoperative treatment were divided into ICU and ward. We referred to these “treatments as the next step” as the admission route.

### 2.4. WBBS

As part of the follow-up plan for multiple-trauma patients, the WBBS did not have an institution-specific protocol; instead, a protocol was conducted at the discretion of the attending physician. The WBBS was performed using Tc-99m methylene diphosphonate (20 mCi) at an average of 10 days (3–21 days) after admission. Images of the regions of interest on WBBS were read by an experienced nuclear medicine physician. Hot-uptake areas in the WBBS were regarded as injured sites and finally diagnosed after additional examinations (such as X-ray or CT scans). During this process, non-active skeletal damage, such as arthritis or old fracture lesions, were classified into the non-missed injury group.

### 2.5. Statistical Analysis

To compare the characteristics of multiple-trauma patients who had missed skeletal injuries and those who did not, the two-sample *t*-test was used for continuous variables, which was based on the normality assumptions derived from the Shapiro–Wilk test. Additionally, chi-square test or Fisher’s exact test were used to compare categorical variables. Univariate and multivariable logistic regression analyses were used to test the association between missed injuries and sex, age, admission department, ISS, GCS, duration from admission to evaluation of the WBBS, admission route, trauma team deployment, OS involvement, shock, and mechanism of injury. The Hosmer and Lemeshow goodness-of-fit test was used to assess the suitability of the models. The discrimination of the model was measured using the concordance index. Analyses were performed using the SAS program (version 9.4; SAS Institute Inc., Cary, NC, USA) and the R Statistical Package (version 3.5.1; Institute for Statistics and Mathematics, Vienna, Austria; www.R-project.org).

## 3. Results

### 3.1. General and Clinical Characteristics

**Figure 1:**
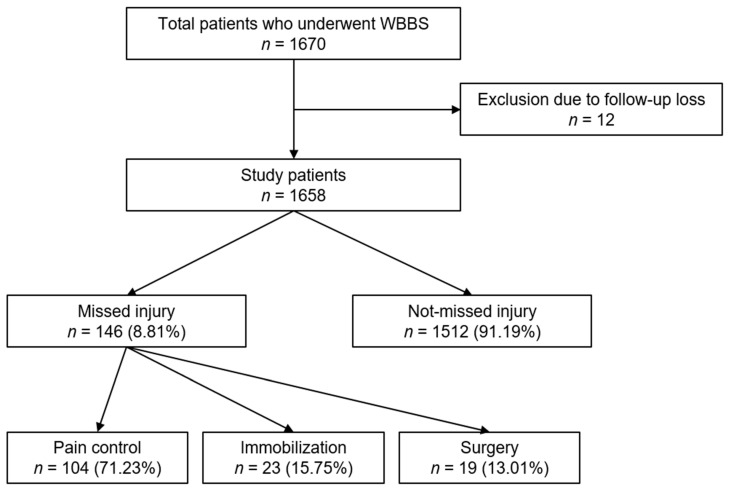
Flow chart of study.

A total of 1670 patients with multiple traumas underwent WBBSs. Twelve patients were excluded because of a loss of follow-up data due to their transfer to the local hospital. Out of the remaining 1658 patients, 146 with missed skeletal injuries were identified. The patient flow chart is summarized in Figure 1. ISS values ≥ 16 were observed in 109 (74.66%) patients in the missed injury group and in 688 (45.50%) patients in the not-missed injury group, and the ISS corresponding to missed skeletal injuries showed a significant difference (*p* < 0.001). The rate of admission through surgery and embolization was high in the missed injury group, and a significant difference was found between the two groups (*p* < 0.001). In addition, the proportion of patients with shock was 19.86%, which was higher in the missed injury group than in the not-missed injury group (*p* < 0.001). A significant difference in OS involvement was also noted between the two groups (*p* = 0.004) (Table 1).

### 3.2. Site of Detected Fracture and Treatment

The most common injury sites in the missed injury group were the chest (86 patients, 58.90%) followed by the upper and lower extremities. Analysis of the treatment pattern in the missed injury group revealed pain control in 104 (71.23%), immobilization in 23 (15.75%), and surgery in 19 (13.01%) patients. As a result of the analysis of the treatment method according to the identified fracture sites, it was determined that pain control was performed for the chest of all patients, surgery was performed most frequently in the upper extremities, and immobilization was performed for the lower extremities (Table 2).

### 3.3. Risk of Missing Skeletal Injuries in Patients with Multiple Traumas According to Clinical Characteristics

As determined through the analysis of the risk of missing a skeletal injury according to clinical characteristics, the odds ratio (OR) was 3.53 times higher (95% confidence interval (CI), 2.40–5.19) in patients with an ISS ≥ 16 than in patients with an ISS < 16. In addition, compared to hospitalization through the intensive care unit, the OR of missed injuries was 1.95 times higher (95% CI, 1.28–2.97) in surgery cases and 4.54 times higher (95% CI, 1.17–17.63) in embolization cases. Moreover, the OR of missed skeletal injuries for patients without OS involvement was 1.66 times higher (95% CI, 1.18–2.34) than it was for those with OS involvement, while the OR of shock patients was 6.82 times higher (95% CI, 4.18–11.14) than those that did not experience shock. The OR for missed skeletal injuries in patients with an ISS ≥ 16, even after adjustment for sex, age, admission route, OS involvement, shock, and mechanism of injury, was 3.98 times higher (95% CI, 2.47–6.41) than that in patients with ISS < 16 (Table 3).

The *p*-values of the Hosmer–Lemeshow Goodness-of-Fit test were >0.05 for the model, indicating that it was suitable. The discrimination ability of the model, measured using the concordance index, was equal to the area under the receiver operating characteristic curve (0.766; Table 3).

### 3.4. Nomogram for the Probability of Missing a Skeletal Injury

**Figure 2:**
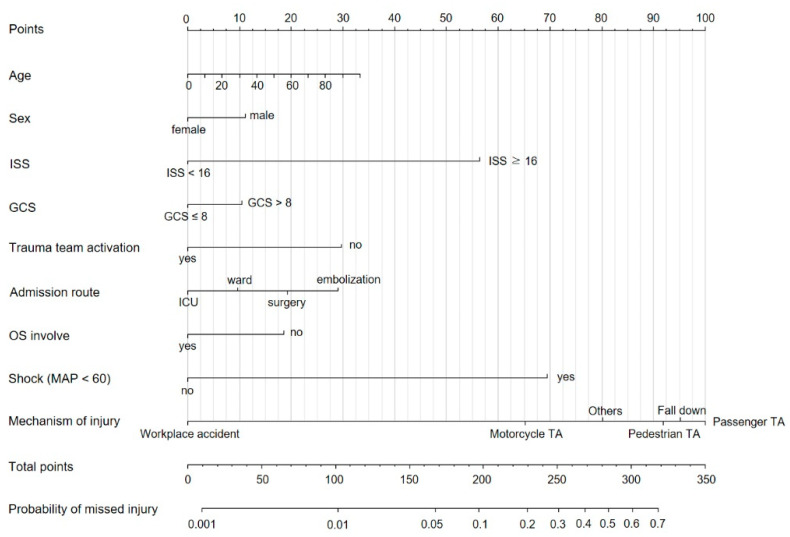
Nomogram for the probabilities of missed injuries.

**Figure 3:**
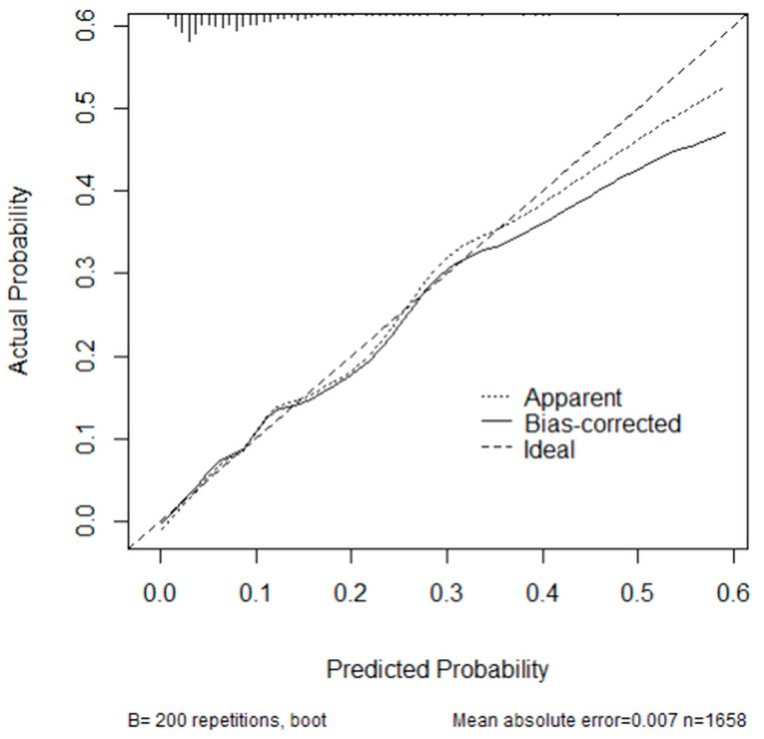
Calibration plot of the nomogram.

A nomogram was constructed based on the multivariable analysis (Figure 2). The nomogram calibration plots indicated good agreement between the predicted and observed outcomes, exhibiting close approximation between the predicted and observed probabilities (Figure 3). The nomogram consists of twelve rows, and the first row (points) contains the point assignment for each variable. Rows 2–10 represent the variables included in the model. For each patient, each variable was assigned a point value based on the patient’s clinical characteristics. To determine the point assignment, a vertical line was drawn between the appropriate variable value and the point line. The assigned points for all nine variables were summed, and the total is presented in row 11 (total points). A vertical line was drawn between the total points and the corresponding value in the final row (the probability of a patient having a missed skeletal injury).

## 4. Discussion

In this study, we reviewed data on trauma patients who had undergone WBBSs and visited the regional trauma center at a tertiary referral center and investigated whether a WBBS is useful for the detection of missed skeletal injuries in this population. Moreover, we evaluated the factors related to missing skeletal injuries. Our study yielded the following main results. First, we identified the statistically significant factors that affect the failure to diagnose a skeletal injury, namely, the ISS, admission route, OS involvement, shock status, and the mechanism of injury. Second, the most common site of a missed skeletal injury was the chest (rib), and the corresponding treatment was different depending on the lesion. In particular, surgery was performed on 13.01% of patients in the missed skeletal injury group. Third, the OR of missed skeletal injuries detected via a WBBS was high in patients with moderate to severe multiple trauma (ISS ≥ 16).

In multiple-trauma patients, the reported incidence of missed injuries varies. Tammelin et al. reported an incidence of 2.7% [5], while Lee et al. reported a 61.8% incidence [8]. In the present study, we evaluated 1658 trauma patients from January 2015 to May 2019, and missed injuries were documented in 146 cases (8.8%). This broad range might be due to large differences in the study populations, study methods, and the definition of missed injuries in the included studies.

The present study was conducted to determine the usefulness of a WBBS as a screening test for missed skeletal injuries in multiple-trauma patients. A bone scan is more sensitive than radiography with respect to detecting bone lesions as it uses physiologic bone changes for imaging [9]. Our results also show that a WBBS is an effective modality for detecting skeletal injuries not found using X-ray or CT scans, especially in patients with moderate to severe trauma. In addition, under the Korean National Health Insurance System, the economic burden can be reduced because a bone scan (USD 90) is cheaper than a CT scan (USD 150, on average) or magnetic resonance imaging (USD 300, on average). The fact that the administered dose of radiation is less than that of CT is also considered to be an advantage of WBBSs [10]. The radiation dose received during a WBBS has been reported to be 6.0 mSv [11]. This is higher than the radiation dose associated with a chest X-ray (0.1 mSv) but is lower than those doses received during a chest CT scan or an abdominal and pelvic CT scan (13.7–15.0 mSv). Bone uptake observed via a WBBS may reveal increased activity for up to 2 years after a fracture. For elderly patients, the results of a WBBS may appear normal even 7–10 days after the injury [8,12]. In the present study, WBBSs were performed for an average of about 10 days (3 to 21 days) after an injury, which is in accordance with the rule that a bone scan can be performed at least 5 days after an injury. The reason WBBSs are extended for up to 21 days after trauma is because early assessments are limited for critically ill patients with a poor general condition.

In previous studies, rib fractures were identified in up to 56% of cases of multiple trauma [8,13,14,15], which is in accordance with our results. The delayed diagnosis of a rib fracture may lead to persistent pain, loss of functional lung capacity, pulmonary complications, and prolonged hospitalization [8,14]. These complications are especially noticeable in patients with severe cases and are associated with increased mortality. The early diagnosis of a rib fracture is necessary to minimize complications and mortality.

Many previous studies have demonstrated that a higher ISS has a significant relationship with missed injuries in trauma patients [16,17,18,19,20]. The ISS is an established medical score used to assess trauma severity and is correlated with mortality, morbidity, and hospitalization duration after trauma. The ISS has been validated in numerous studies; this has become one of the most common scoring systems used to assess injured patients in trauma outcome research [21,22]. In our study, the ISS score was also found to be significant according to the multivariate analysis with respect to missed injuries. Since it is very difficult to find missed fractures after the initial assessment of multiple-trauma patients, constant efforts are needed to reduce these missed injuries.

Understanding the mechanism of trauma is necessary to obtain valuable clues for the diagnosis of missed injuries in trauma patients. Fitschen-Oestern et al. reported that missed foot injuries were common among patients who were involved in car accidents or patients who fell from a great height [23]. Furnival et al. reported that an injury resulting from an automotive accident was a significant predictor of a patient having a missed injury [24]. In our analysis of the mechanism of injury, we did not find a significant difference between the two groups. However, in the univariate analysis, the OR for missed skeletal injuries was significantly lower in workplace accidents than in car accidents. Therefore, patients with blunt trauma, especially when sustained through a car accident, should be carefully evaluated.

Hensgens et al. have reported the missed injury rate after the inter-hospital transfer of severely injured trauma patients [25]. They mentioned that GCS was the only significant predictable risk factor for missed injuries, which contrasts with the findings of our study. The different structure of their study group might explain the difference in the two sets of results. Moreover, the effect of GCS on missed skeletal injuries might be biased by the initial GCS measurement error, shock, or extreme pain. The GCS is a reliable neurological indicator for assessing a patient’s state of consciousness. It was initially used for the evaluation of head trauma patients but is now applied to all acute medical and trauma patients. The deterioration of a patient’s consciousness can lead to missed diagnoses during an initial physical examination or history documentation. Although GCS has not been identified as a significant factor in the present study, it should be fully considered in clinical practice.

The nomogram derived from our study was verified through its calibration plot (Figure 1 and Figure 2). Among the variables affecting the probability of missing a skeletal injury, the mechanism of injury was the most influential, followed by the ISS and shock. Considering each variable, if the total score exceeds 300, there is a 60% probability of a patient having a missed skeletal injury. Therefore, considering the variables and scores reflected in this nomogram, we recommend performing a WBBS when there is a high probability of missing a skeletal injury.

Another method of minimizing missed injuries is the use of a trauma tertiary survey [26]. According to Enderson et al., a tertiary survey was conducted to reduce the frequency of missed injuries [2]. A tertiary survey is a simple and easy approach used to detect undiagnosed injuries in trauma patients. It consists of re-evaluating patients 24 h after admission with history documentation, physical examination, a review of previous examinations, and the performance of new examinations if needed. In particular, like the results of this study, the initial evaluation might have been neglected in patients who were in shock or who underwent an intervention such as surgery or embolization. Therefore, in such a situation, a tertiary survey should also be carefully considered. Likewise, if OS was involved in the initial assessment, a tertiary survey could be of great help in the diagnosis. If, according to the procedure, a tertiary survey was conducted without problems, it could be an effective and easily applicable approach to identifying missed injuries on admission [27]. Even though tertiary surveys were also conducted at our institution, 146 of the missed injuries found during the study period are thought to have been due to limitations in the physical examination of patients, especially critically ill patients. In particular, among patients with analgesics or sedatives administered in the ICU, there was a limit to detecting missed injuries through physical examination, and the additional examination, i.e., the next step, did not proceed. In this respect, a WBBS can help avoid missing injuries and can be considered a test for a tertiary survey.

This study has several limitations. First, this study potentially suffered from selection bias because it is not a randomized retrospective analysis performed by a single institution. Second, our study population was not large enough to generalize the results. Third, since this study was conducted using the Korean single-region trauma center’s trauma team deployment criteria, WBBS protocol, and insurance criteria, the results of this study may have limited ability to be generalized to other institutions or countries. Fourth, we exclusively focused on skeletal injuries and did not consider missed injuries other than bone damage, which can lead to different results and misinterpretations that might not be present in other studies that address missed injuries. Therefore, our findings should not be generalized to other trauma patients, and systemic multicenter randomized prospective trials are needed to validate our findings. However, despite these limitations, this study could be the impetus for further research in the field of trauma and may help cast the use of a WBBS in a more positive light.

## 5. Conclusions

In conclusion, missed skeletal injuries were significantly associated with several statistical factors, and a WBBS is a screening method that can detect missed skeletal injuries in patients with multiple traumas. Furthermore, systemic multicenter prospective studies are needed to confirm the efficiency and validity of WBBSs.

## Figures and Tables

**Table 1:** Characteristics of multiple-trauma patients according to missed skeletal injuries.

	Total (*n* = 1658)	Missed Injury (*n* = 146)	Not-Missed Injury (*n* = 1512)	*p*-Value
Gender				0.269
Male	1160 (69.96)	108 (73.97)	1052 (69.58)	
Female	498 (30.04)	38 (26.03)	460 (30.42)	
Age (mean ± SD)	57.06 ± 18.17	58.47 ± 15.40	56.93 ± 18.41	0.260
Admission department				0.069
NS	1062 (64.05)	100 (68.49)	962 (63.62)	
GS	261 (15.74)	29 (19.86)	232 (15.34)	
TS	204 (12.30)	9 (6.16)	195 (12.90)	
OS	123 (7.42)	8 (5.48)	115 (7.61)	
Others	8 (0.48)	-	8 (0.53)	
ISS				<0.001
ISS < 16	861 (51.93)	37 (25.34)	824 (54.40)	
ISS ≥ 16	797 (48.07)	109 (74.66)	688 (45.50)	
GCS				0.090
GCS ≤ 8	1487 (89.69)	125 (85.62)	1362 (90.08)	
GCS > 8	171 (10.31)	21 (14.38)	150 (9.92)	
Duration ^a^ (day)	10.68 ± 4.10	10.58 ± 5.09	9.59 ± 4.36	0.396
Admission route				<0.001
ICU	681 (41.07)	52 (35.62)	629 (41.60)	
Ward	635 (38.30)	45 (30.82)	590 (39.02)	
Surgery	331 (19.96)	46 (31.51)	285 (18.85)	
Embolization	11 (0.66)	3 (2.05)	8 (0.53)	
Trauma team deployment				0.321
Yes	1194 (72.01)	100 (68.49)	1094 (72.35)	
No	464 (27.99)	46 (31.51)	418 (27.65)	
OS involvement				0.004
Yes	1100 (66.34)	81 (55.48)	1019 (67.39)	
No	558 (33.66)	65 (44.52)	493 (32.61)	
Shock ^b^				<0.001
Yes	82 (4.95)	29 (19.86)	53 (3.51)	
No	1576 (95.05)	117 (80.14)	1459 (96.49)	
Mechanism of injury				0.107
Passenger TA	570 (36.14)	57 (40.71)	513 (35.70)	
Motorcycle TA	15 (0.95)	2 (1.43)	13 (0.90)	
Pedestrian TA	255 (16.17)	21 (15.00)	234 (16.28)	
Workplace accident	76 (4.82)	1 (0.71)	75 (5.22)	
Fall	286 (18.14)	31 (22.14)	255 (17.75)	
Others ^c^	375 (23.78)	28 (20.00)	347 (24.15)	

SD—standard deviation, NS—neurosurgery, GS—general surgery, TS—thoracic surgery, OS—orthopedic surgery, ISS—injury severity score, GCS—Glasgow coma scale, ICU—intensive care unit, and TA—traffic accident. ^a^ Duration from admission to evaluation of the whole-body bone scan. ^b^ Mean arterial pressure <60, v/s unstable. ^c^ Assault, rolling down, slipping down, and stab wound.

**Table 2:** Site of detected injury and treatment in missed injury group.

Site of Detected Injury	Total (*n* = 146)	Treatment (*n* = 146)		
		Pain Control (*n* = 104)	Immobilization (*n* = 23)	Surgery (*n* = 19)
Face				
Mandible	1 (0.68)	-	-	1 (5.26)
Chest				
Rib	84 (57.53)	84 (80.77)	-	-
Sternum	2 (1.37	2 (1.92)	-	-
Upper extremity				
Clavicle	11 (7.53)	4 (3.84)	2 (8.70)	5 (26.32)
Scapula	9 (6.16)	2 (1.92)	6 (26.09)	1 (5.26)
Humerus	1 (0.68)	-	-	1 (5.26)
Radius	2 (1.37)	1 (0.96)	1 (4.35)	-
Hand	10 (6.85)	2 (1.92)	-	8 (42.11)
Lower extremity				
Pelvis	1 (0.68)	1 (0.96)	-	-
Ankle	4 (2.74)	-	3 (13.04)	1 (5.26)
Tibia	4 (2.74)	1 (0.96)	2 (8.70)	1 (5.26)
Fibula	8 (5.48)	1 (0.96)	6 (26.09)	1 (5.26)
Foot	5 (3.42)	4 (3.84)	1 (4.35)	
Spine				
T-spine	2 (1.37)	-	2 (8.70)	-
L-spine	2 (1.37)	2 (1.92)	-	-

T-spine—thoracic spine; L-spine—lumbar spine.

**Table 3:** Result of univariate and multivariable logistic regression analyses testing the relationship between missed injuries and clinical characteristics.

	Univariate		Multivariable	
	Odds Ratio (95% CI)	*p*-Value	Odds Ratio (95% CI)	*p*-Value
Gender				
Female	reference		reference	
Male	1.24 (0.85–1.83)	0.269	1.25 (0.82–1.91)	0.304
Age	1.01 (1.00–1.01)	0.329	1.01 (1.00–1.02)	0.110
Admission department				
OS	reference			
NS	1.49 (0.71–3.15)	0.979		
GS	1.80 (0.80–4.06)	0.977		
TS	0.66 (0.25–1.77)	0.985		
Others	NA	NA		
ISS				
ISS < 16	reference		reference	
ISS ≥ 16	3.53 (2.40–5.19)	<0.001	3.98 (2.47–6.41)	<0.001
GCS				
GCS ≤ 8	reference		reference	
GCS > 8	1.53 (0.93–2.50)	0.093	1.25 (0.69–2.26)	0.471
Duration ^a^ (day)	1.00 (0.99–1.01)	0.479		
Admission route				
ICU	reference		reference	
Ward	0.92 (0.61–1.40)	0.724	1.53 (0.92–2.55)	0.115
Surgery	1.95 (1.28–2.97)	0.001	1.77 (1.11–2.85)	0.026
Embolization	4.54 (1.17–17.63)	0.022	1.98 (0.35–11.38)	0.440
Trauma team deployment				
Yes	reference		reference	
No	1.20 (0.83–1.74)	0.322	2.30 (1.18–4.49)	0.015
OS involvement				
Yes	reference		reference	
No	1.66 (1.18–2.34)	0.004	1.46 (0.86–2.47)	0.160
Shock ^b^				
No	reference		reference	
Yes	6.82 (4.18–11.14)	<0.001	5.90 (3.26–10.68)	<0.001
Mechanism of injury				
Passenger TA	reference		reference	
Motorcycle TA	1.39 (0.31–6.29)	0.289	0.40 (0.08–2.08)	0.167
Pedestrian TA	0.81 (0.48–1.36)	0.580	0.81 (0.47–1.41)	0.391
Workplace accident	0.12 (0.01–0.88)	0.040	0.08 (0.01–0.61)	0.002
Fall	1.09 (0.69–1.74)	0.085	0.89 (0.54–1.46)	0.716
Others ^c^	0.73 (0.45–1.17)	0.846	0.61 (0.35–1.06)	0.096
Hosmer and Lemeshow Goodness-of-Fit test			0.240	
C-statistics			0.766	

CI—confidence interval, OS—orthopedic surgery, NS—neurosurgery, GS—general surgery, TS—thoracic surgery, ISS—injury severity score, GCS—Glasgow coma scale, ICU—intensive care unit, TA—traffic accident, and NA—not available. ^a^ Duration from admission to evaluation using the whole-body bone scan. ^b^ Mean arterial pressure <60; v/s unstable. ^c^ Assault, rolling down, slipping down, and stab wound.

## Data Availability

The data presented in this study are available on request made to the corresponding author. The data are not publicly available due to privacy of enrolled patients.
